# Structurally related odorant ligands of the olfactory receptor OR51E2 differentially promote metastasis emergence and tumor growth

**DOI:** 10.18632/oncotarget.13836

**Published:** 2016-12-09

**Authors:** Guenhaël Sanz, Isabelle Leray, Denise Grébert, Sharmilee Antoine, Adrien Acquistapace, Adeline Muscat, Abdelhak Boukadiri, Lluis M. Mir

**Affiliations:** ^1^ NBO, INRA, Université Paris-Saclay, 78350, Jouy-en-Josas, France; ^2^ Vectorologie et thérapeutiques anti-cancéreuses, UMR8203, CNRS, Univ. Paris-Sud, Université Paris-Saclay, Gustave Roussy, Villejuif, France; ^3^ GABI, AgroParisTech, INRA, Université Paris-Saclay, 78350, Jouy-en-Josas, France

**Keywords:** olfactory receptors, odorant ligands, tumor progression, prostate cancer

## Abstract

Olfactory receptors are G protein-coupled receptors. Some of them are expressed in tumor cells, such as the OR51E2 receptor overexpressed in LNCaP prostate cancer cells. It is considered a prostate tumor marker. We previously demonstrated that this receptor is able to promote LNCaP cell invasiveness *in vitro* upon stimulation with its odorant agonist β-ionone, leading to increased generation of metastases *in vivo*. In the present study, we show that even a relatively short exposure to β-ionone is sufficient to promote metastasis emergence. Moreover, α-ionone, considered an OR51E2 antagonist, in fact promotes prostate tumor growth *in vivo*. The combination of α-ionone with β-ionone triggers a higher increase in the total tumor burden than each molecule alone. To support the *in vivo* results, we demonstrate *in vitro* that α-ionone is a real agonist of OR51E2, mainly sustaining LNCaP cell growth, while β-ionone mainly promotes cell invasiveness. So, while structurally close, α-ionone and β-ionone appear to induce different cellular effects, both leading to increased tumor aggressiveness. This behaviour could be explained by a different coupling to downstream effectors, as it has been reported for the so-called biased ligands of other G protein-coupled receptors.

## INTRODUCTION

Olfactory receptors (ORs) are mainly known to be expressed in the olfactory sensory neurons (OSNs) of the olfactory epithelium where they permit to detect and discriminate myriads of diverse odorant molecules. These receptors are G protein-coupled receptors (GPCRs) encoded by an exceptionally large multigene family [1, 2]. Besides their function in odorant sensing, ORs play additional roles, both in the olfactory epithelium, where they are involved in OSN sorting and targeting to the olfactory bulb [3, 4], and in non-olfactory tissues. For instance, it has been reported that ORs drive chemotaxis in sperm [5, 6], promote cell migration and adhesion in muscle [7] and increase serotonin secretion by enterochromaffin cells in the gut [8, 9] or by pulmonary neuroendocrine cells in lungs [10]. ORs were also reported to induce renin release in the kidney and raise in blood pressure in response to fermentation products (short chain fatty acids) of the gut microbiota [11, 12]. In lungs, pulmonary macrophages express ORs with a potential role in the response to microbial infection, by allowing bacterial metabolite detection by macrophages, which leads to their migration to the infection site [13]. It has also been speculated that some ORs expressed in eyes participate in sensing chemicals and maintain eye homeostasis [14]. Of particular interest was the demonstration that some ORs are overexpressed in various tumors and are considered tumor biomarkers [15–19]. Noteworthy, a study reported the overexpression of 34 ORs genes in breast tumors of CHEK2 1100delC-mutation carriers and concluded that the OR protein family could have a role in cancer progression [20]. Another recent publication pointed out that the olfactory transduction pathway appears to be significantly associated with increased pancreatic cancer risk [21]. It was also reported in non-cancerous cells but also in cancer cell lines that ORs can favor cell division by participating in early cytokinesis, exerting a regulatory role on actin cytoskeleton [22]. Finally, our recent publication [23] demonstrated the involvement of ORs in tumor progression: we showed, for the first time, that the PSGR (Prostate Specific G protein-coupled Receptor, also named OR51E2), an OR endogenously expressed in LNCaP prostate cancer cells, promotes cell invasiveness *in vitro* and metatasis emergence *in vivo*. We also showed that a PI3 kinase γ (PI3K γ) dependent signaling pathway is involved in this process. Interestingly, a few months later, another group published that the PSGR plays a role in the regulation of chronic inflammation and in the initiation of prostate pathogenesis through the regulation of NF-κB and a PI3K/AKT signaling pathway [24]. The same group also recently reported that PSGR overexpression synergizes with loss of PTEN to accelerate prostate cancer development [25].

In the present study, we further explored the involvement of the PSGR in prostate cancer progression and tried to counteract its promoting effect on metastasis occurrence and dissemination using α-ionone, an odorant molecule reported as a PSGR antagonist [26]. For this purpose the model described in our previous study was used again, namely the LNCaP cells subcutaneously inoculated in Nod Scid Gamma (NSG) mice, stimulated with β-ionone, a PSGR agonist [23]. We also investigated the effect of the duration of the β-ionone treatment. While our results confirmed that β-ionone stimulation of LNCaP cells promotes their ability to generate metastases *in vivo*, surprisingly, they also revealed that α-ionone effects were not those expected. Therefore we also investigated *in vitro* the effects of α-ionone on LNCaP cells.

## RESULTS AND DISCUSSION

### *In vivo* growth of LNCaP prostate tumors and metastasis emergence upon various OR51E2 odorant ligand stimulation

**Figure 1:**
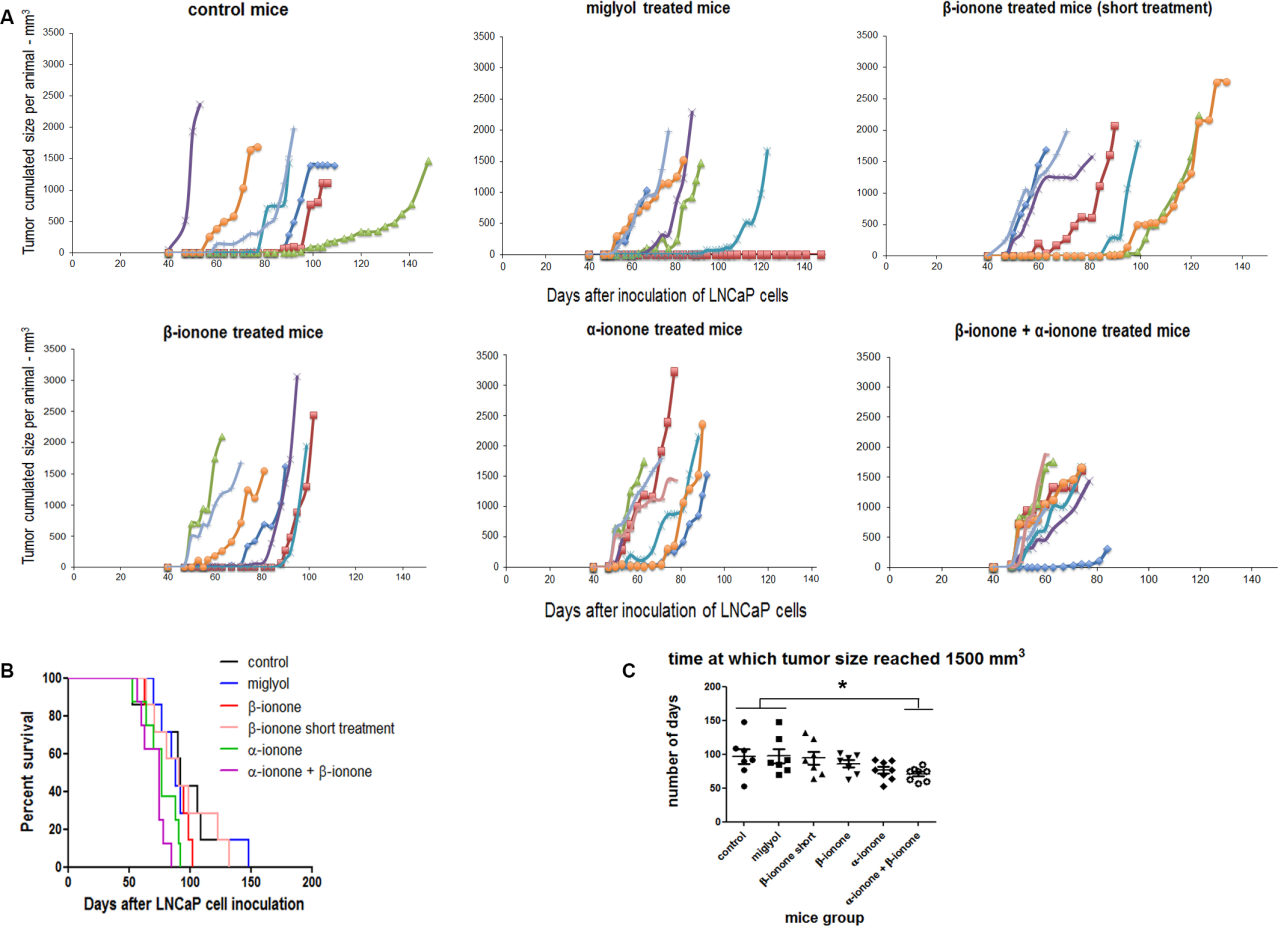
*In vivo* tumor growth upon exposure of mice to odorant ligands of the OR51E2 receptor (**A**) LNCaP cells were subcutaneously implanted in NSG mice and tumor size was measured with a caliper. In each panel, corresponding to the various mice groups that received different treatments, the sum of the volumes of the tumors and inguinal nodes measured for each animal was presented as a function of the time elapsed since LNCaP cell inoculation. Mice were treated (twice a day during the first six weeks and then three times a week) by applying on their skin: Miglyol (a neutral oil that was used to dilute odorants), 1 mM β-ionone diluted in Miglyol, 2 mM α-ionone diluted in Miglyol or a mixture of 1 mM β-ionone and 2 mM α-ionone diluted in Milgyol. A group of mice were also treated with 1 mM β-ionone for a shorter duration (twice a day during two weeks). The mice of the control group were not treated. Each mouse of a group is represented by a color. (**B**) Kaplan-Meier analysis comparing the different mice groups. Mice were sacrificed as soon as the volume of one tumor or node reached 1500 mm^3^. (**C**) Days after LNCaP cell inoculation for which the size of one of the tumors (or inguinal nodes) reached 1500 mm^3^. For each mice group, data normality was checked using the Kolmogorov-Smirnov test. Then, statistics were performed using a two-tailed Student's test (* for *p* ≤ 0.05).

**Figure 2:**
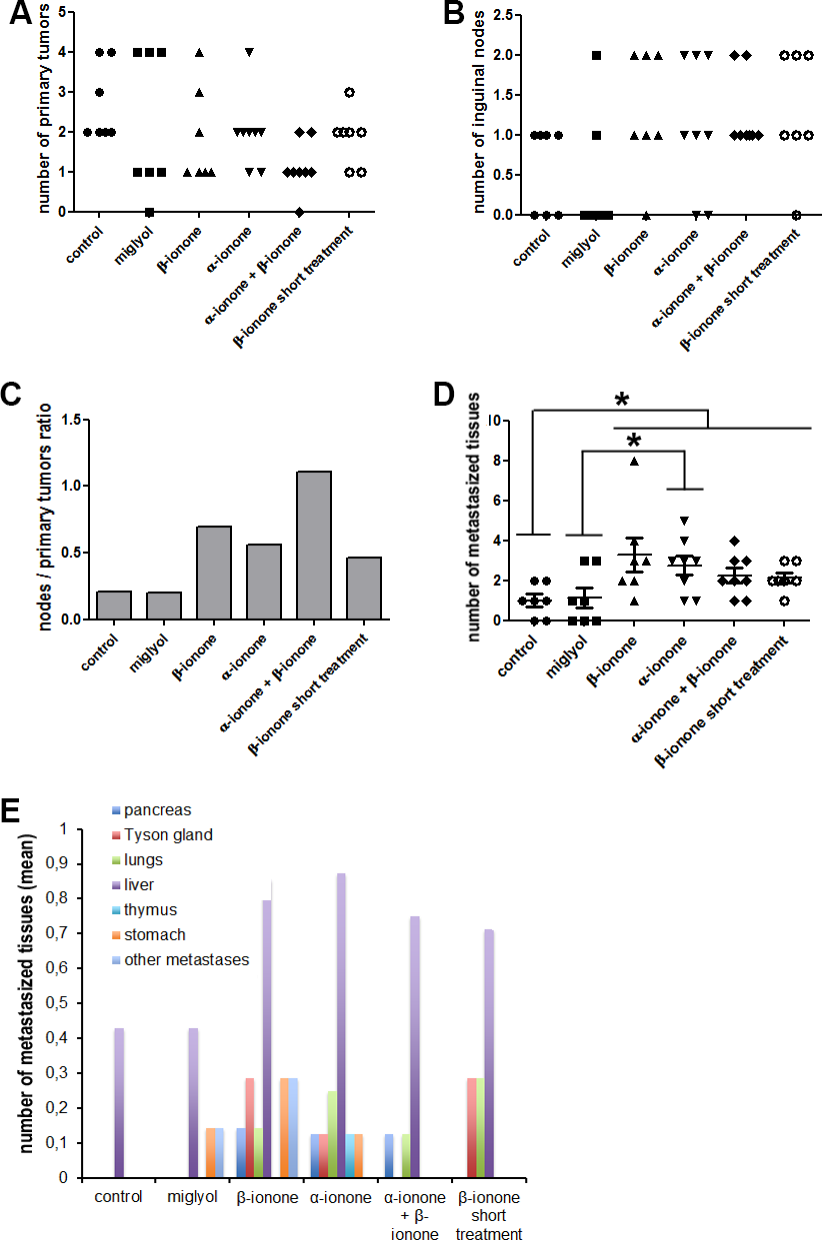
Metastasis emergence upon exposure to odorant ligands of the OR51E2 receptor LNCaP cells were subcutaneously implanted in NSG mice. Mice were treated (twice a day during the first six weeks and then three times a week) by applying on their skin: Miglyol (a neutral oil that was used to dilute odorants), 1 mM β-ionone diluted in Miglyol, 2 mM α-ionone diluted in Miglyol or a mixture of 1 mM β-ionone and 2 mM α-ionone diluted in Milgyol. A group of mice were also treated with 1 mM β-ionone for a shorter duration (twice a day during two weeks). The mice of the control group were not treated. For ethical reasons, mice were sacrificed as soon as one of the tumors, nodes or metastasis of the mice reached 1500 mm^3^. The primary tumors, inguinal nodes and various tissues were collected during autopsy. Metastases originating from inoculated LNCaP cells were searched by HES staining and immunohistochemistry using anti-PSA and anti-PSGR antibodies. (**A**) Numbers of primary tumors. (**B**) Number of invaded inguinal nodes in each mouse for each mice group. (**C**) The ratio of the number of nodes to the number of primary tumors is also specified. (**D**) Cumulated number of metastasized tissues in each animal of each group. (**E**) Mean number of metastasized tissues for each mice group in function of the tissue. For each mice group, data normality was checked using the Kolmogorov-Smirnov test. Then, statistics were performed using a two-tailed Student's test (* for *p* ≤ 0.05).

The LNCaP prostate cancer cells express the OR51E2 receptor, and β-ionone and α-ionone were reported to act as an agonist and an antagonist of this receptor respectively. As in our previous published study [23], we used castrated male mice in order to avoid the stimulation of the androgen receptor also expressed in LNCaP cells, as well as the potential stimulation of the OR51E2 receptor by androgens (some androgens were reported to be OR51E2 ligands by Neuhaus *et al*. [26]). Furthermore, using male castrated mice allowed us to obtain results that could be relevant for understanding the androgen-independent progression of prostate cancer. Besides, after we started the present *in vivo* study, it was reported that short chain fatty acids (SCFA) could also activate the OR51E2 receptor [12]. So, since we did not control the level of SCFA in our experiment, it could happen that those newly identified OR51E2 ligands could explain a part of the interindividual variability that we observed. Mice inoculated subcutaneously with LNCaP cells were treated (as described in the Materials and Methods section) with β-ionone, α-ionone or a mixture of these odorants in order to investigate the potential ability of α-ionone to counteract the effects induced by β-ionone on tumor progression. Neuhaus *et al*. reported in [26] the EC_50_ of the OR51E2 receptor for the β-ionone ligand using heterologous expression of OR51E2 in HEK 293 cells and calcium imaging. This EC_50_ is about 2.5 μM and the plateau starts at 50 μM β-ionone. However, in other *in vitro* experiments, the same authors used β-ionone at concentrations of 250 μM or 500 μM on LNCaP cells to activate the OR51E2 receptor. So, since we do not know how much odorant molecules reached LNCaP cells *in vivo* after their application to the skin, we assumed that applying 1mM β-ionone to the skin should allow sufficient amounts of the compound to reach the cells. Moreover, performing calcium imaging on LNCaP cells transfected with siRNAs targeting the OR51E2 receptor, Neuhaus *et al*. showed that 500 μM β-ionone directly applied on LNCaP cells only triggered a calcium response in the presence of OR51E2. Thus, assuming that a part of the amount of β-ionone applied to the skin would not reach LNCaP cells, we speculated that 1mM β-ionone applied to the skin would mainly induce effects on LNCaP cells through OR51E2 activation. We also added 100 μM β-ionone to the Matrigel used to inoculate LNCaP cells, since we knew that this concentration was efficient to promote LNCaP cell invasiveness *in vitro* [23]. Regarding the concentrations of α-ionone, we used twice more than β-ionone since Neuhaus *et al*. reported an antagonist effect of α-ionone at a ratio 2:1, and we also showed in [23] that α-ionone was able to counteract β-ionone-induced LNCaP cell invasiveness at this ratio. Consequently, we spread 2 mM α-ionone on mice skin and added 200 μM to the Matrigel. The controls included groups of mice either untreated or treated with Miglyol, the neutral oil used to dilute odorants and helping their penetration through the skin. Contrary to the mineral oil, the uncompletely characterized mixture that we used in our initial study [23] and that slightly increased LNCaP cell motility, the Miglyol was devoid of any effect on LNCaP cell motility. Beside, a group of mice was treated with β-ionone for a shorter duration (twice a day during two weeks for this short treatment compared to twice a day during the first six weeks and then three times a week until sacrifice for the longer treatment) in order to know whether β-ionone effects could be relevant after a relatively short treatment or required a prolonged one. We followed the tumor growth at the sites of cell inoculation, as well as the growth of lymph nodes invaded by the cancer cells. Different end-points were used to evaluate the effects of the two compounds *in vivo*: the cumulated cancerous tissues volume (tumors and nodes) as a function of the time after cell inoculation (Figure 1), and the number of metastasized tissues at the time of the mice sacrifice which was dictated by ethical reasons (sacrifices were performed as soon as the volume of one of the tumors or nodes reached 1500 mm^3^) (Figure 2). As shown in Figure 1, β-ionone did not significantly accelerate tumor growth. Unexpectedly, the comparison of the curves reporting mice survival in the different groups suggests that α-ionone, the reported OR51E2 antagonist, accelerated tumor growth (because tumors reached the 1500 mm^3^ size faster and mice had to be euthanized earlier), while no statistical significance could be reached due to a too low number of animals in each group. This effect seemed even more pronounced when α-ionone was combined with β-ionone. On Figure 1C, the pairwise comparison of the groups in terms of tumor growth rate, shows that α-ionone combined with β-ionone provoked a significant acceleration of the tumor growth.

Regarding the effect of the extended treatment with β-ionone, we confirmed the results of our previous study [23] : even if in the present study we used Miglyol instead of mineral oil, β-ionone showed again a clear tendency to promote LNCaP cell invasion of inguinal nodes and metastasis dissemination in various tissues (Figure 2). Unfortunately, no statistical significant conclusion could be drawn due to the too small number of animals and to some variability of the individual responses in each group (yet, the probability that the number of metastasized tissues is increased in the β-ionone treated mice compared with the miglyol treated mice is equal to 0.054, very close of the significance). Interestingly, whereas β-ionone was applied at a lower dose than α-ionone and did not promote tumor growth alone, it enhanced metastasis emergence as much as α-ionone. This could be due to the fact that β-ionone would mainly promote LNCaP cell invasiveness and therefore metastasis dissemination whereas α-ionone would mainly promote LNCaP cell proliferation and tumor growth, which can also lead to metastasis appearence (this hypothesis was later supported by the results obtained in the *in vitro* experiments described below). Moreover, we also observed that a relatively short treatment with β-ionone (two weeks) was already able to promote inguinal node settlement by LNCaP cells and metastasis emergence in other tissues, but to a lesser extent than a longer treatment. Thus, *in vivo* stimulation of LNCaP cells with β-ionone seems to rapidly provoke the dissemination of the cells, and the increase in the duration of the stimulation appears to sustain LNCaP cell spreading in the body.

Surprisingly, while both α-ionone and β-ionone separately promoted metastasis emergence, the results presented in Figure 2D and 2E (and Supplementary Figure S1) show that the mixture of β-ionone and α-ionone tended to induce less metastases than each molecule alone. Nevertheless, we can observe in Figure 2A, 2B and 2C that the treatment with the mixture led to the highest number of inguinal nodes invaded by LNCaP cells and to the lowest tumor engraftment despite the induction of the fastest tumor growth (Figure 1). This suggests that the mixture of β-ionone and α-ionone promoted the largest migration of LNCaP cells to inguinal nodes at the expense of tumor engraftment at the inoculation sites. Furthermore, the mice treated with both odorants had to be sacrificed earlier due to a faster increase in tumor burden (tumors at the cell inoculation sites and inguinal nodes). This can explain why the number of metastasized tissues (Figure 2D and 2E) and their variety (Figure 2E and Supplementary Figure S1) were less important upon stimulation with both odorants (while LNCaP cell migration to inguinal nodes (Figure 2B and 2C) was higher).

### *In vitro* growth and invasiveness of LNCaP cancer cells upon various OR51E2 odorant ligand stimulation

**Figure 3:**
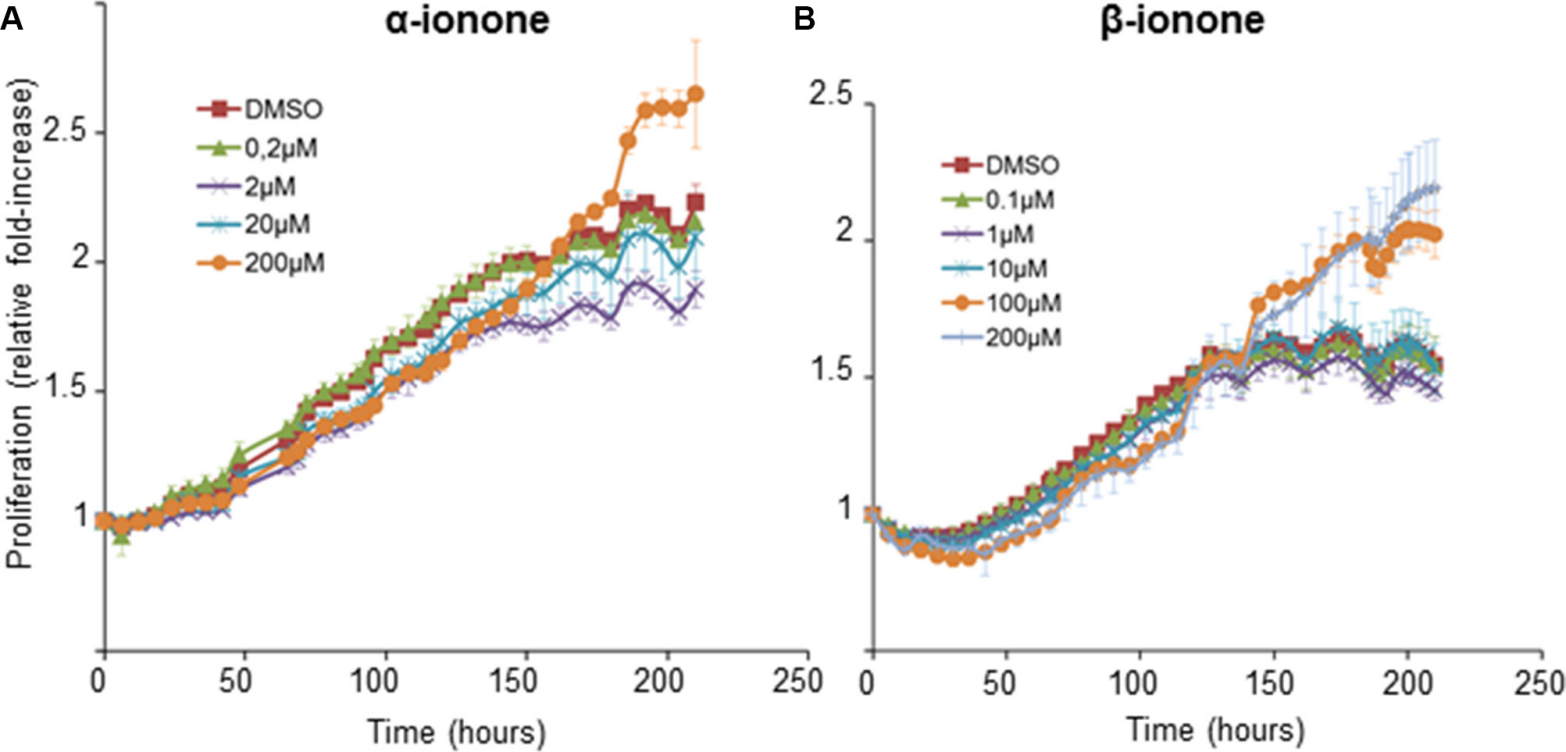
LNCaP cell growth induced by α-ionone or β-ionone LNCaP cells were seeded onto a collagen I gel in the presence of various concentrations of (**A**) α-ionone (0.2, 2, 20 or 200 μM) or (**B**) β-ionone (0.1, 1, 10, 100 or 200 μM), or of 0.1% DMSO (the amount of DMSO used to dilute odorants before adding them to the collagen gel or culture medium). Cell confluence was measured for 9 days and results are presented as an increase of proliferation during time (using proliferation=1 at t_0_). Bars indicate standard deviation (*n* = 3).

Because the concentration (2 mM) of the α-ionone solution used to treat mice was twice the concentration of the β-ionone solution, the effect of α-ionone on tumor growth could be either specifically linked to this odorant molecule or simply due to its larger concentration. Thus we investigated *in vitro* LNCaP cell proliferation upon α-ionone or β-ionone stimulation (Figure 3 and Supplementary Figure S2). Neuhaus *et al*. reported that the EC_50_ of the OR51E2 receptor for the β-ionone ligand was about 2.5 μM using OR51E2 expression in HEK 293 cells and calcium imaging. Also, performing other *in vitro* experiments on LNCaP cells, the same authors used β-ionone at 250 or 500 μM. So, since the β-ionone EC_50_ appeared to be a few micromolar in a given experimental context while β-ionone was used at concentrations hundred times higher (and even more) in other experimental contexts, for our *in vitro* experiments, we chose to test concentrations from the micromolar to hundreds of micromolar. LNCaP cell proliferation appeared to be prolonged upon stimulation with either α- or β-ionone. Indeed, in the presence of the highest concentrations of α-ionone or β-ionone, cells continued to proliferate for about 40 hours after the LNCaP cells stopped their proliferation in the control conditions and at low or moderate doses of α-ionone or β-ionone. This overgrowth suggests that the highest tested doses of α-ionone or β-ionone could alleviate cell contact inhibition which could sustain LNCaP cell proliferation *in vitro*. Since *in vitro* stimulations of LNCaP cells with 200 μM α-ionone induced effects similar to those generated by 100 μM α-ionone (Supplementary Figure S2) or by 100 and 200 μM β-ionone (Figure 3), the faster tumor burden increase observed *in vivo* (Figure 1C) in the presence of α-ionone (and not in the presence of β-ionone alone) could be mainly due to the higher efficacy of that molecule to enhance tumor growth, rather than to the use of a dose twice larger.

**Figure 4:**
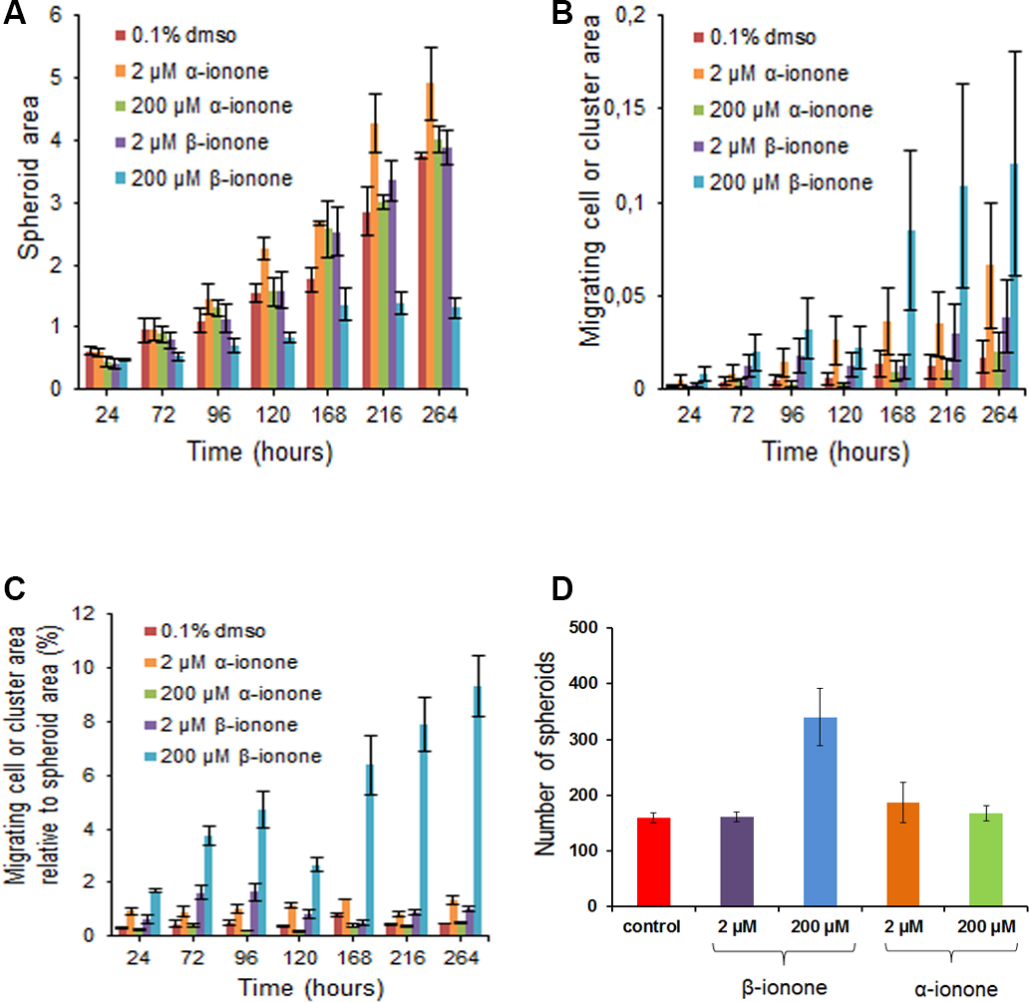
Effects of α-ionone or β-ionone on spheroid growth and cell migration Spheroids obtained from LNCaP cells were grown into a collagen I gel in the presence of various concentrations of α-ionone (2 or 200 μM) or β-ionone (2 or 200 μM), or of 0.1% DMSO (the amount of DMSO used to dilute odorants before adding them to the collagen gel or culture medium). The areas occupied by spheroids or by migrating cells (which were either single or clustered cells) were quantified (number of pixels) using the ImageJ software. The migrating cell clusters are differentiated from the spheroids given that there were not present at the time of spheroid seeding in the collagen gel: actually, these clusters appeared near the primary spheroid periphery for the duration of the experiment. At the time of initial plating in collagen, the number of spheroids was about the same for each condition tested. At the end of the experiment, the number of spheroids was counted to evaluate the apparition of secondary spheroids that could be due to the growth of the migrating clustered cells. Bars indicate standard deviation (*n* = 3). (**A**) Spheroid area over time. (**B**) Migrating cell area over time. (**C**) Migrating cell area relative to spheroid area over time. (**D**) Number of spheroids after ten days of growth.

In line with this idea, the results of spheroid growth upon odorant exposure show that a moderate concentration of α-ionone (2 μM) was able to increase spheroid growth while β-ionone was not (Figure 4A). Surprisingly, in apparent contradiction with the effects observed regarding LNCaP cell growth, 200 μM α-ionone had no effect on spheroid growth, while 200 μM β-ionone slowed down spheroid growth and blocked its growth at day 168 (Figure 4A). These discrepancies between these two assays could be the result of a different cell behavior within a spheroid and within a monolayer and/or the result of a different accessibility of the odorants to cells. The balance between cell proliferation at spheroid periphery, cell death inside spheroids and cell migration from spheroids can all contribute to the different effects of the odorants in spheroids compared to what happens in cell monolayers. Part of these differences can be explained by the induction of cell migration from spheroids caused by 200 μM β-ionone (Figure 4B and 4C). Indeed, this migration can compete with spheroid growth. This conclusion is in agreement with the fact that the number of spheroids per well was increased, and the spheroid size reduced, in the presence of 200 μM β-ionone compared to control conditions or conditions for which cell migration was not greatly increased (Figure 4D). Such a phenomenon cannot be assessed on cells growing in monolayers. The ratio of the migrating cells to the spheroid growth further emphasizes the significant spreading of the cells in the presence of 2 μM of either β-ionone or α-ionone, as well as the massive spreading in the presence of 200 μM β-ionone (Figure 4C).

### α-ionone is an agonist of OR51E2 in LNCaP cells with complex effects on growth and invasiveness

**Figure 5:**
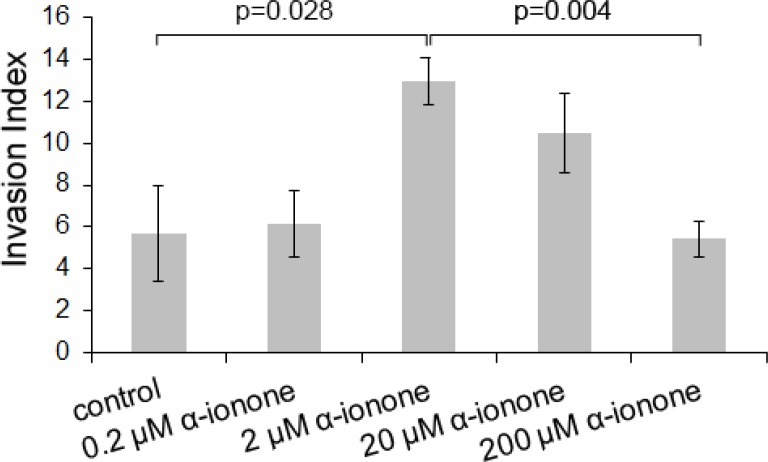
LNCaP cell invasiveness induced by α-ionone LNCaP cell invasiveness was assessed on collagen I gel in the presence of various concentrations of α-ionone (0.2, 2, 20 or 200 μM) or of 0.1% DMSO (the amount of DMSO used to dilute α-ionone before adding it to the collagen gel or culture medium). The invasion index corresponds to the percentage of cells presenting invasive extensions into the collagen gel 24 hours after seeding. Bars indicate standard deviation (*n* = 3). Statistics were performed using a two-tailed Student's test.

**Figure 6:**
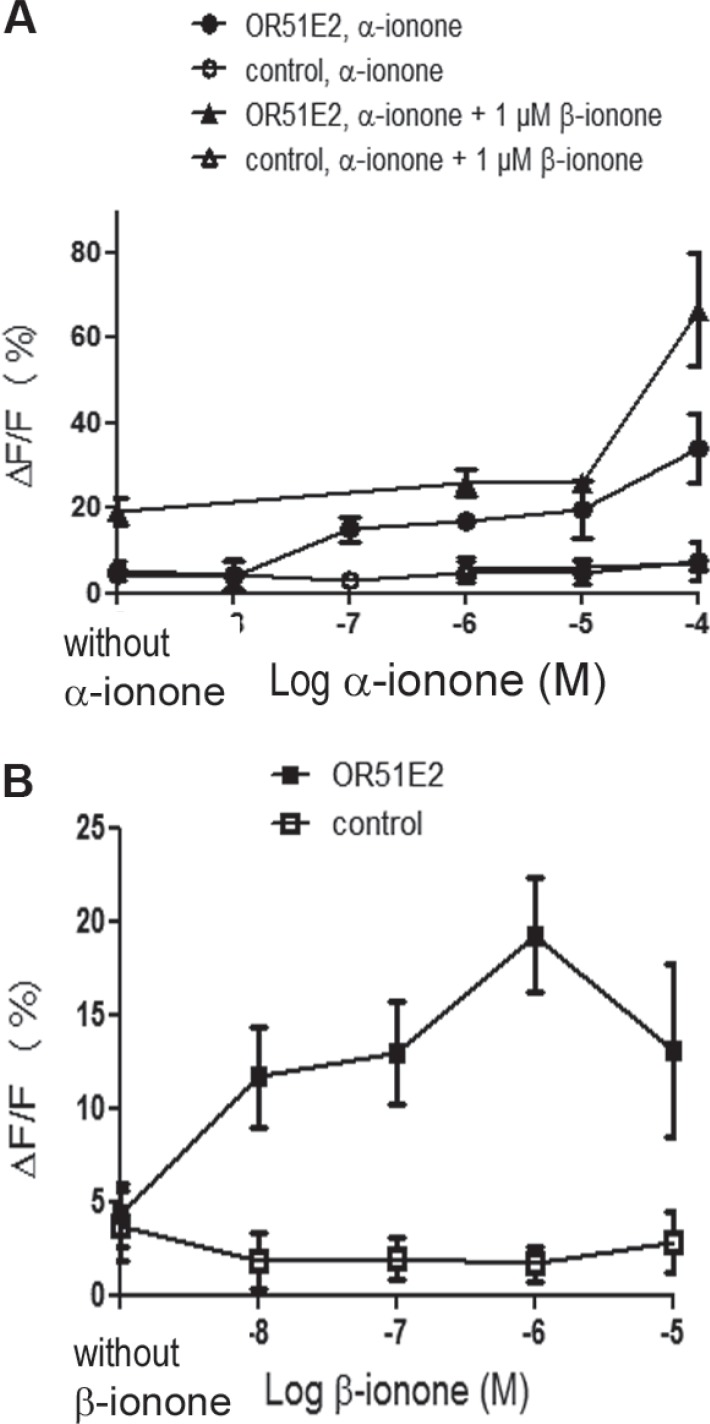
Calcium increase induced by α-ionone, β-ionone or mixtures of α-ionone and β-ionone on HEK293 cells transiently transfected with OR51E2 HEK 293 cells were transiently transfected to express OR51E2 or mock-transfected (control). 72 h later, cells were loaded with the calcium sensitive dye Fluo-4 and stimulated with (**A**) α-ionone (10^−8^ to 10^−4^ M), mixed or not with 1 μM β-ionone or (**B**) β-ionone (10^−8^ to 10^−5^ M). β-ionone was also applied at 10^−4^ M, but the response of control cells was near the response of OR51E2 expressing cells (control cells: ΔF/F = 13.77 ± 5.5 %; OR51E2 expressing cells: ΔF/F = 16.84 ± 3 %), so this point is not shown due to lack of specificity. Yet, the response induced by 10^−4^ M β-ionone on OR51E2 expressing cells is not higher than the one induced by 10^−6^ M β-ionone. Calcium responses due to the interaction between the OR and odorant molecules are expressed as the mean fluorescence variation ΔF/F (%). (filled symbols) OR51E2 cells; (open symbols) control cells; bars indicate standard deviation (*n* ≥ 3).

While α-ionone seemed to accelerate tumor growth *in vivo* (Figure 1), we also observed that, with respect to the controls, α-ionone treatment of mice induced an increase in the number of inguinal nodes invaded by LNCaP cells as well as an increase in the number of metastases originating from LNCaP cells in other tissues, as shown in Figure 2. We searched to determine whether this promoting effect of α-ionone on metastasis emergence was due to the faster tumor growth induced by α-ionone (as suggested by the *in vitro* effects of the highest α-ionone concentration, Figure 3) and/or to a potential promotion of LNCaP cell invasiveness (as suggested by the *in vitro* effects of a lower α-ionone concentration, Figure 4). The latter was assessed on collagen I gels in the presence of various α-ionone doses. As shown in Figure 5, 2 μM α-ionone significantly increased LNCaP cell invasiveness, whereas higher α-ionone doses had a weaker effect or even no effect at all. In a previous study we demonstrated that 200 μM α-ionone inhibited β-ionone induced LNCaP cell invasiveness [23]. So, our results suggest that that moderate doses of α-ionone could activate the OR51E2 receptor and induce cell invasiveness while higher α-ionone doses (e.g. 200 μM) would inactivate this receptor. This is in agreement with the reported bell-shaped dose response curve of ORs that can be explained by the inactivation of ORs at high ligand doses [27]. Yet, since 200 μM α-ionone induced a sustained LNCaP cell proliferation (Figure 3), another explanation could be that different signaling pathways would be induced depending on the α-ionone doses: they would trigger different outcomes such as higher cell invasiveness or increased cell proliferation. In line with this idea, using HEK 293 cells transiently expressing OR51E2 and their counterpart that did not express OR51E2, we demonstrated that α-ionone can activate OR51E2 leading to an increase in the cytosolic calcium at 100 μM (Figure 6A). Furthermore, we also showed that α-ionone was not able to inhibit the optimal calcium response induced by 1 μM β-ionone on HEK293 cells transiently expressing OR51E2, even using one hundred times more α-ionone than β-ionone (Figure 6A and 6B). So, by this mean, we confirmed that α-ionone is a real PSGR agonist and we demonstrated that OR51E2 was not inhibited by high α-ionone doses neighboring 100 μM. Furthermore, on Figure 6A, it can be noticed a kind of plateau in the calcium response induced by α-ionone between 10^−7^ and 10^−5^ M and then, at 10^−4^ M, an increased efficacy of α-ionone to trigger intracellular calcium release. Thus, all of these results support that OR51E2 probably induces various signaling pathways depending on the α-ionone concentration.

All these hypotheses regarding the effect of α-ionone on the OR51E2 receptor remains to be further explored in future studies. In any case, our results demonstrate that α-ionone is not a mere OR51E2 antagonist as previously reported [26]. Moreover, we can observe that the dose response curve induced by β-ionone on HEK 293 cells expressing OR51E2 differs from that of α-ionone, showing a bell-shaped appearence. This leads to assume that α-ionone and β-ionone do not activate the OR51E2 receptor similarly and could trigger different outcomes. At last, the calcium increase induced by 100 μM α-ionone seems to be enhanced by the co-application of 1 μM β-ionone, reaching a level that exceeds the addition of the levels induced by each molecule alone at the same concentration. While the dose response to α-ionone and β-ionone is not the same in the cell growth assay of Figure 3, such a synergistic effect, not necessarily obtained at the same ratio of α-ionone and β-ionone, could explain why the *in vivo* tumor growth was even more accelerated upon the exposure to α-ionone mixed with β-ionone compared to the exposure to α-ionone alone.

## MATERIALS AND METHODS

### Ethics statement

The animals were handled in conformity with the Guidelines of the French government regarding operative procedures and animal care. Protocol was approved by the ethics committee for experiments with animals CEEA-26 (“Comité d’Ethique en Expérimentation Animale n ° 26”) (protocol number 2012–043).

### Chemicals

Odorants and DMSO were purchased from Sigma-Aldrich, Fluka or Acros Organics at the highest purity available. Miglyol 812N was purchased from Cremer Oleo Division. Paraffin (CellWax) was obtained from CML, and hemalun, eosin, and safran from RAL.

### Mammalian expression vectors

OR51E2 coding sequence was amplified from human genomic DNA, sequenced (Beckman Coulter Genomics) and inserted into the pCMV-Tag3B mammalian expression vector (Stratagene). The resulting vector was named pCMV-TagOR51E2. The OR51E2 cloned sequence is the same as the GenBank sequence AF311306.

### Cell culture and transfection

LNCaP cells were purchased from ATCC (Clone FGC, No. CRL-1740TM) at passage 19, and grown in RPMI 1640 medium (ATCC) supplemented with 10% fetal bovine serum (ATCC), at 37°C in a humidified incubator with 5% CO_2_.

Human embryo kidney 293 (HEK 293) were grown in MEM culture medium without phenol red (Gibco) supplemented with 10% fetal bovine serum, at 37°C in a humidified incubator with 5% CO_2_. These cells were transiently transfected with pCMV-TagOR51E2 using lipofectamine 2000 (Invitrogen) according to the manufacturer's instructions.

### *In vitro* assessment of cell invasion

Collagen type I gels were prepared as described by De Wever [28]. Cells were cultured for 48 hours before seeding them as a suspension of single cells deposited on top of the collagen type I gels at a density of 10^4^ cells/cm^2^. To stimulate ORs, odorants were first diluted into DMSO and then into the collagen I solution or culture medium. For controls, DMSO was added at the same final concentration used to dilute odorants (0.1%). 24 hours after cell seeding, invasive cells presenting invasive extensions into the collagen gel and non-invading cells were counted in 10–15 microscope fields randomly selected. Results were expressed as the percentage of invasive cells (invasion index).

### *In vitro* assessment of cell proliferation

Collagen type I gels were prepared as for cell invasion assays. 250 μL of collagen gel were polymerized at the bottom of wells of a 24-well culture plate. 6.10^4^ LNCaP cells were seeded on the collagen gel in 400 μL of culture medium per well. Odorants were added to the collagen gel and culture medium. For this purpose, odorants were first diluted into DMSO at a concentration of 0.1, 0.2, 1, 2, 10, 20, 50, 100 or 200 mM. These odorant solutions were then diluted 1000 fold in the collagen gel or culture medium to reach a final concentration of 0.1, 0.2, 1, 2, 10, 20, 50, 100 or 200 μM. As a control, the same amount of DMSO (0.1%) used to dilute odorants was added to the collagen gel and the culture medium. The culture medium, with the corresponding concentration of the odorant, was renewed daily for 9 days and cell confluence was measured by an IncuCyte ZOOM instrument (Essen Bioscience). Each condition was tested in three wells. To avoid cross-contamination of the odorants, experiments using α-ionone or β-ionone were performed independently.

### *In vitro* assessment of spheroid growth and cell invasion

LNCaP cells were grown in a 24-well Ultra Low Attachment cell culture plate (Costar) (10 000 cells were seeded in each well). The culture medium was renewed every day. After four days of culture, spheroids were transfered in a collagen I gel according to the protocol described in [28]. Briefly, a collagen I gel was prepared and supplemented with β-ionone, α-ionone, or the amount of DMSO used to dilute odorants (0.1%). 500 μL of collagen gel were distributed per well of a 12-well cell culture plate and gelification was performed 1 hour in the incubator. The spheroids were collected in a 15 mL Falcon tube and left to sediment by the gravitational pull. The supernatant was removed and spheroids were mixed in a proper volume of collagen I gel supplemented with β-ionone, α-ionone or DMSO. 500 μL of spheroid suspension were distributed onto the existing collagen layer. After 1 hour in the incubator, 1 mL of culture medium containing β-ionone, α-ionone or DMSO was added onto the collagen layers. This medium was renewed daily for 15 days. Spheroids were followed over time by photographing them. The areas occupied by spheroids, released cells or clusters were measured using ImageJ.

### Mice

Nod Scid Gamma (NSG) male mice were bred in the animal housing facilities of the Institut Gustave Roussy, with free access to food and water. Plastic cages were connected to controlled ventilated racks. The cages with the animals exposed to the odorants were connected to a separated ventilation unit.

### *In vivo* assessment of cell invasion and metastases

LNCaP cells at passage 25 were inoculated into 8 week-old castrated male NSG mice (castration was performed two weeks before cell inoculation). 10^6^ cells were suspended in 50 μL of RPMI 1640 plus 50 μL of Matrigel (BD Biosciences) and injected subcutaneously with a 26G needle at 2 sites in each flank of the mice. Cells were inoculated in Matrigel (to retain the cells as much as possible at the injection site) in the presence of the respective odorants diluted in DMSO. Depending on the ulterior treatment by β-ionone and/or α-ionone, we correspondingly added 100 μM β-ionone or 200 μM α-ionone to the Matrigel since we previously demonstrated that 100 μM β-ionone induced LNCaP cell invasiveness and 200 μM α-ionone was able to counteract this β-ionone effect [23]. After inoculation, the skin above the inoculation sites was regularly exposed to the odorants diluted in Miglyol to facilitate their penetration across the skin. Since we do not know how much odorant molecules reached the inoculated LNCaP cells, we assumed that ionone doses in the millimolar range should allow sufficient amounts of the compound to reach the cells. We thus applied 1mM β-ionone on mice skin, and 2 mM α-ionone in order to respect the 2:1 ratio that was reported to assure the α-ionone antagonist effect [26]. The odorants were spread with a sterile cotton swab dipped in the respective odorant-containing Miglyol solution.

More precisely, to inoculate the cells in the presence of odorants, β-ionone was first diluted into DMSO at a concentration of 100 mM and then 1000 fold into the cell-containing RPMI + Matrigel mixture. Similarly, α-ionone was first diluted into DMSO at a concentration of 200 mM and then into the RPMI + Matrigel mixture at the final concentration of 200 μM. β-ionone 100 mM was also mixed with α-ionone 200 mM in DMSO and the mixture was diluted 1000 fold into the cell-containing RPMI + Matrigel mixture. For the control groups without odorant stimulation, DMSO (0.1% final concentration) was also added to the cell-containing RPMI + Matrigel mixture. A first group of 7 mice was inoculated with LNCaP cells (in the presence of DMSO) and received no further treatment. Seven other mice were inoculated with LNCaP cells (in the presence of DMSO) and Miglyol was spread on the skin as described here above. This treatment was repeated twice a day during six weeks and then three times a week until sacrifice. The other groups of mice (7 or 8 animals per group) were inoculated with LNCaP cells in the presence of β-ionone, α-ionone or both odorants. These mice were respectively treated with 1 mM β-ionone, 2 mM α-ionone, or a mixture of 1 mM β-ionone and 2 mM α-ionone, directly diluted in Miglyol, twice a day during six weeks and then three times a week until sacrifice. A last group of 7 mice was inoculated with LNCaP cells in the presence of β-ionone and treated only twice a day during two weeks with 1 mM β-ionone directly diluted in Miglyol. Tumor size was measured with a caliper and tumor volume was calculated as 0.5 x length x width squared. For ethical reasons, mice were sacrificed as soon as the volume of one of the tumors, nodes or metastases reached 1500 mm^3^. Upon autopsy, tumors and various tissues such as lymph nodes, lungs, liver, Tyson glands, stomach, pancreas and thymus were sampled. Tissues were fixed for 24 hours in formaldehyde then stored in 70% ethanol at 4°C. All samples were dehydrated in ethanol and included in paraffin. Serial sections of 5 μm thickness were prepared and dewaxed in toluene and rehydrated in ethanol and then water. Some sections were stained with hemalun (RAL), eosin (RAL) and safran (RAL) (HES staining). Immunohistochemistry was performed on other sections using anti-PSGR (LS-A6332, Cliniscience), anti-PSA (ab9537, abcam), or rabbit serum as a negative control, the Vectastain Elite ABC-Peroxidase Kits Rabbit IgG (Cliniscience), and a DAB revelation kit (SK-4100, Vector).

### Calcium imaging

Calcium imaging experiments were performed as described in [23]. HEK293 cells were seeded onto a 96-well culture plate (black microtiter plate, Greiner Bio-one) at a density of 0.9.10^5^ cells per well. 24 hours later, cells were loaded with 2.5 μM of Fluo-4 acetoxymethyl ester (Molecular Probes). Calcium imaging was performed using an inverted epifluorescence microscope (CK40 Olympus) equipped with a digital camera (ORCA-ER, Hamamatsu Photonics). Ca^2+^ reponses were observed at 460–490 nm excitation and ≥ 515 nm emission wavelengths. Data acquisition and analysis were performed using the SimplePCI software (Hamamatsu, Compix). The odorant α-ionone was prepared extemporaneously by a first dilution into DMSO and then serial dilutions into Hanks’ salt solution (Eurobio) supplemented with 20 mM Hepes, pH 7.2. Stimulations were performed at concentrations that do not elicit significant calcium responses in mock-transfected cells. The Ca^2+^ signal was measured as the relative change in fluorescence intensity ΔF/F = (F-F_0_)/F_0_, where F_0_ is the fluorescence level before stimulation. Results were expressed as the mean of the ΔF/F of at least twenty cells.

## CONCLUSIONS

In the present study, we confirm that the PSGR agonist β-ionone promotes LNCaP prostate cancer cell invasiveness and propensity to generate metastases. The fact that an OR can promote tumor cell invasiveness and migration is in line with the previous publications reporting OR involvement in the migration of various cell types such as olfactory sensory neurons, sperm, muscle cells and macrophages [3, 5–7, 13]. Furthermore, we demonstrate that a relatively short exposure to β-ionone is sufficient to promote metastasis spreading and that a sustained exposure amplifies this effect. Given that β-ionone is present in food, beverages and cosmetics, this result might be of interest regarding potential prevention practices for prostate cancer patients. In particular, our results could be interesting regarding prostate cancer progression in an androgen-independent context. Indeed, the aggressiveness of prostate tumor cells expressing the OR51E2 receptor could increase due to their exposure to ionones and independently of androgen stimulation of the androgen receptor or the OR51E2 receptor.

In addition, our results demonstrate that α-ionone is not a PSGR antagonist as described by Neuhaus et al. [26]. Our initial hypothesis was that α-ionone could counteract β-ionone effects *in vivo*. On the contrary, using various approaches, we demonstrate that α-ionone behaves like a real PSGR agonist. Yet, α-ionone does not induce exactly the same effects that β-ionone: α-ionone appears to mainly promote tumor cell growth while β-ionone mainly promotes tumor cell invasiveness. This suggests that the signal transduction pathways induced by α-ionone and β-ionone would be different, which could be explained by a different coupling to G proteins or β-arrestins, as it has been reported for the so-called biased ligands of other GPCRs [29]. A recent study also described that an OR expressed in a mouse OSN can trigger various signaling pathways depending on the ligand which interacts with it [30]. We plan to explore that question in future studies.

Moreover, α-ionone seems to induce different effects depending on its concentration, from cell motility increase at moderate concentrations (one to ten micromolar), which appears to moderately activate the OR51E2 receptor (Figure 6A), to cell growth promotion at higher concentrations (at least one hundred micromolar) for which the receptor appeared more efficiently activated (Figure 6A). *In vivo*, this balance would be between tumor cell invasiveness and tumor and metastasis growth. Especially, *in vitro*, the highest doses of α-ionone tested do not induce cell invasiveness while moderate doses do: this could be due to inactivation of the OR51E2 receptor at high ligand doses or to activation of an alternative signaling pathway that would not trigger cell invasiveness. The present demonstration that α-ionone is able to activate the OR51E2 receptor heterologously expressed in HEK 293 cells at 100 μM supports the latter hypothesis. Moreover, high doses of α-ionone appeared to allow sustaining LNCaP cell growth: in this case, an alternative route induced at these doses would permit to alleviate cell contact inhibition. Further studies to decipher the signaling pathways involved upon exposure to various doses of α-ionone should allow clarifying the meaning of these results. Yet, the α-ionone dose response observed on Figure 6A suggests that α-ionone-induced signaling pathways could depend on the partial or full activation of the receptor, or in other words to different active conformations of the receptor, which could result in different interactions with the downstream signaling partners. Nevertheless, since it is known that a same odorant molecule can activate different ORs with different efficacies, we cannot exclude that α-ionone could activate another OR that could be expressed in LNCaP cells and that our observations on the various effects of α-ionone depending on its dose would be the result of the involvement of different receptors at moderate or higher doses. To ascertain that the ionone effects that we presently describe are only due to OR51E2 activation, we should downregulate the OR51E2 expression in LNCaP cells. Yet, trying to knockdown the OR51E2 receptor in LNCaP cells using siRNAs, the cells stopped to grow 48 hours after siRNA transfection compared with the control cells (untransfected or transfected with a control scrambled siRNA), increased in size (as senescent cells) and died. So, for the moment, it has been impossible to perform the same experiments (*in vitro* or *in vivo*) using LNCaP cells that would not express the OR51E2 receptor. Therefore, further studies to investigate the role of the OR51E2 receptor in LNCaP cell survival and growth would also be of great interest.

## SUPPLEMENTARY FIGURES


