# Supplementary material for: Structurally related odorant ligands of the olfactory receptor OR51E2 differentially promote metastasis emergence and tumor growth
Source: Oncotarget. 2016 Dec 9;8(3):4330–41. doi: 10.18632/oncotarget.13836 (PMC5354835; doi:10.18632/oncotarget.13836)
Supplement: Supplementary file 1 [file oncotarget-08-4330-s001.pdf]

Structurally related odorant ligands of the olfactory receptor OR51E2 differentially promote metastasis emergence and tumor growth

Supplementary Materials

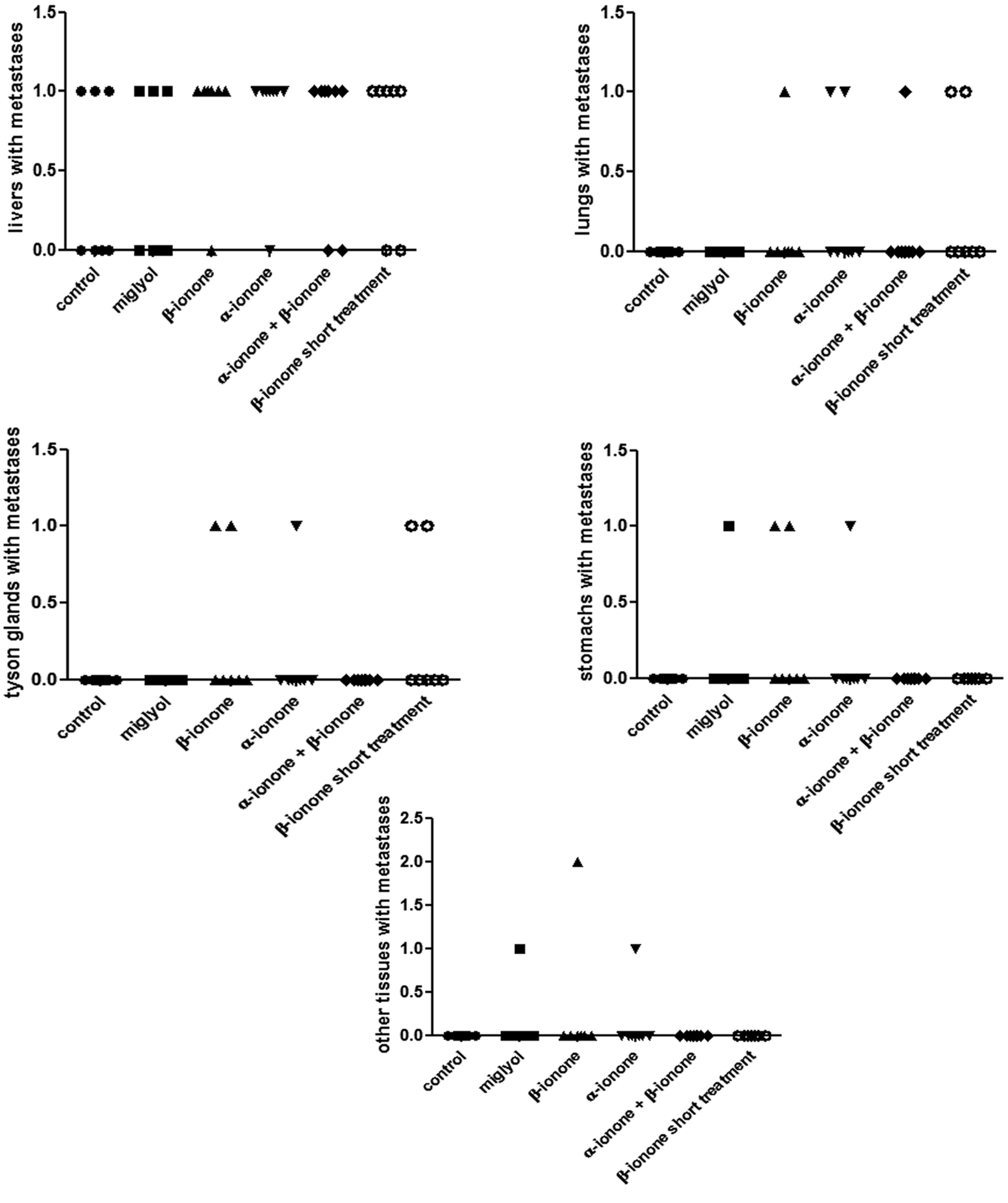

Supplementary Figure S1: Metastasized tissues in each mouse upon exposure to odorant ligands of the OR51E2 receptor. LNCaP cells were subcutaneously implanted in NSG mice. Mice were treated (twice a day during the first six weeks and then three times a week) by applying on their skin: Miglyol (a neutral oil that was used to dilute odorants), 1 mM β-ionone diluted in Miglyol, 2 mM α-ionone diluted in Miglyol or a mixture of 1 mM β-ionone and 2 mM α-ionone diluted in Milgyol. A group of mice were also treated with 1 mM β-ionone for a shorter duration (twice a day during two weeks). The mice of the control group were not treated. Metastases originating from inoculated LNCaP cells were searched by HES staining and immunohistochemistry using anti-PSA and anti-PSGR antibodies. The number of metastasized tissues in each animal of each group is shown.

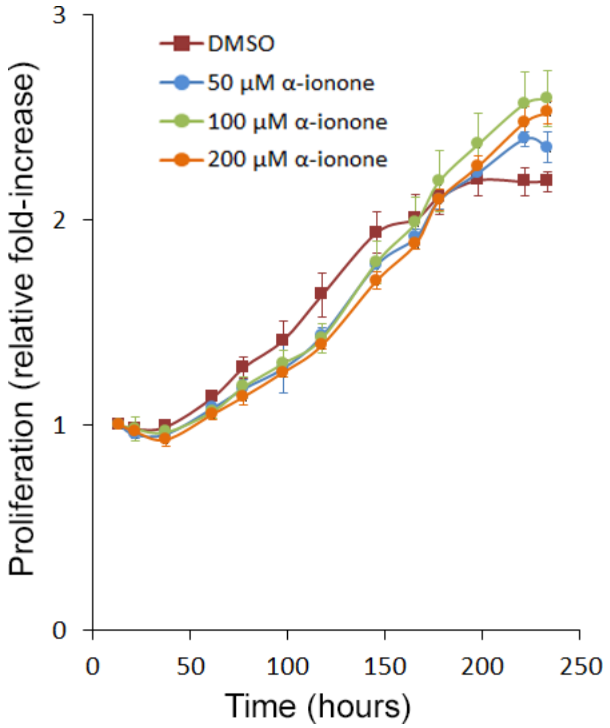

Supplementary Figure S2: LNCaP cell growth induced by α-ionone. LNCaP cells were seeded onto a collagen I gel in the presence of various concentrations of α-ionone (50, 100 or 200 μM) or of 0.1% DMSO (the amount of DMSO used to dilute α-ionone before adding it to the collagen gel or culture medium). Cell confluence was measured for 9 days and results are presented as an increase of proliferation during time (using proliferation=1 at t_0). Bars indicate standard deviation (*n* = 3).
